# Demographical domains and clinico-radiological characteristics of study cohorts with simultaneous multiple intracerebral hemorrhages in a tertiary neurosurgical center in Nepal: a cross-sectional study

**DOI:** 10.12688/f1000research.108816.1

**Published:** 2022-02-07

**Authors:** Sunil Munakomi, Dipak Chaulagain

**Affiliations:** 1 Neurosurgery, College of Medical Sciences, Chitwan, Nepal; 2 Neurosurgery, Uzhhorod National University, Uzhhorod, Ukraine

**Keywords:** multiple strokes, simultaneous strokes, cause, patterns, pathogenesis, outcome

## Abstract

**Background:** Spontaneous simultaneous multiple intra-cerebral hemorrhages (SMICHs) and its occurrences in different territories of arterial disposition has been viewed as uncommon clinical occurrences, since the pathophysiological and predisposing factors as mechanisms aren’t vividly defined.

This research primarily aims for demographic stratification and dichotomization pertaining to risk factors, etiological classifications, anatomical distributions and outcome analysis by focusing on management strategies and pertinent stroke care.

**Methods: **40 patients presenting to the College of Medical Sciences, Chitwan, Nepal in the last two years were included in the study. The patients with two-or-more spontaneous SMICHs with affected arterial territories with similar tomographic density based profiling were chosen as samples. Regression analysis was chosen to test three hypotheses.

**Results:** Among our study cohorts, cortical and cortical territory (60%) was the major anatomical patterns of involvement. A conservative approach was undertaken in nine patients (22.5%), whereas surgical intervention was needed in five others (12.5%). A total of 14(35%) patients leaving against medical advice and a further seven (17.5%) patients were referred for adjuvant oncologic care. Mortality was observed among five (12.5%) patients. Hypertension was seen as a significant variable in its pathogenesis. Male patients were more affected. Age groups comprising of 36-45years and 56-65 years were involved in 32.5% and 30% cases respectively.

**Conclusion:** This study proves the need for a national stroke data bank pertaining to spontaneous SMICHs. This will help foster effective patient education during preoperative counselling; as well as formatting a management algorithm combating them.

## Introduction

Simultaneous multiple intra-cerebral hemorrhages (SMICHs) are characterized by intracerebral hemorrhages within the two-or-more distinct and non-contiguous intracranial vascular territories visualized in the initial radiological imaging ( Wu *et al.*, 2017). Generally intracerebral hemorrhage (ICH) connotes high odds of continuum morbidity and mortality ( Laiwattana *et al.*, 2014). Among ICH patients, approximately 5% (1:20) have been identified as SMICHs ( Renard *et al.*, 2020).

Stroke ripples affect patients with continuum multi-spectral consequences ( Gokce *et al.*, 2016). Literature scarcity pertaining to SMICH paves the way for further research on the entity. Better knowledge and in-depth information pertaining to the incidence and patterns of SMICHs can help formulate treatment algorithms as well as provide newer reforms in patient management system.

### Hypotheses

The research focused on pattern analysis on how the patients with SMICH are affected by applying age and gender as demographic variable and “hypertension” as the major risk variable.

Thus, three hypotheses were formulated:

– ***Hypothesis 1:*** There exists an imperative association between age and SMICH;
– ***Hypothesis 2:*** There exists an imperative association between gender and SMICH;
– ***Hypothesis 3:*** There exists an imperative association between the hypertension and SMICH.

### Rationale of study

The primary aim was to examine risk factors in SMICH patients:

– To analyze the demographic profile (age and gender)of SMICH patients;
– To analyze pivotal factors that impact SMICH patients significantly, namely: hypertension, diabetes, etiological classifications, anatomical distributions and outcome analysis.

## Methods

The methodology for the research was focused to SMICH patients from the hospital medical record section of the College of Medical Sciences, in Bharatpur, Chitwan, Nepal, presenting between January 2019 to January 2021 (time we started recording and compiling data of patients with SMICHs). The outcomes were compiled to study the patterns through frequencies measures and analysis. The approval for the research conduction was obtained by the Institutional Review Committee (COMSTH-IRC/2021–56). Written informed consent was obtained from all the participants or their next of kin (in case their poor Glasgow coma scale did not allow taking consent from the patients themselves) at the time of their admissions.

### Theoretical framework

The research mainly focused upon the SMICH patients as the independent variable and how it impacts the respondents under varied dependent variables like: age, gender and hypertension.

### Inclusion and exclusion criteria

40 patients from the College of Medical Sciences, Chitwan, Nepal with SMICH were included in this study. Data was retrieved from the medical record section of the hospital pertaining to patients presenting with SMICHs. The study size was arrived through convenience sampling.

Inclusion criteria

- All patients presenting with SMICHs.

Exclusion criteria include:

- History of previous strokes;
- Patients with recurrent strokes;
- Failure to obtain consent for participation in the study.

### Target and sampling

The minimal necessary sampling for research has been estimated by Fischer’s formula ( Charan & Biwas, 2013) as


N=a2xsxr/m2

Where:

***a*** represents a value of *1.96* at the confidence-interval of 95%,

***s*** represents SMICH prevalence as 6%,

***r*** as *1-s*and

***m*** represents the margin-of-error as 10%.

The final result through sampling estimation was calculated as 21.66 and the sample-size of40patientswas finalized for the research purpose.

### Variables under focus

SMICH is the presence of two-or-more ICHs that affects different vascular territories with similar tomographic density-profiles. All findings were validated with radiologists.

### Data collection

All data was taken from patient medical records.

Etiologic classification for the cause of ICH was stratified as per the SMASH-U classification into hypertensive angiopathy, cerebral amyloid angiopathy, structural vascular abnormalities, medication related, other systemic causes, and undefined causes respectively ( Mosconi *et al.*, 2021). The risk factors were defined as:

1. Arterial hypertension if blood pressure was above 160/90 mm Hg for more than two readings at the time of admission, on antihypertensive drugs or previous medical history of hypertension.
2. Diabetes mellitus if fasting glucose level was >120 mg/dL at the time of admission; hypercholesterolemia, and fasting cholesterol level >220 mg/dL.
3. Vascular lesion with pertinent radiological imaging at the site of the ICH was classified as the primary cause of the hematoma.
4. Cerebral venous sinus thrombosis was diagnosed on a cerebral venogram.
5. Drug associated ICH was considered in a patient on warfarin with international normalized ratio ≥2.0, novel oral anticoagulants within three days, full-dose heparin, or on systemic thrombolysis.
6. Cerebral amyloid angiopathy was classified in patients’ ≥55 years of age with predominant lobar bleeds with microbleeds on magnetic resonance imaging (MRI) sequences sparing the basal ganglion and the thalamus.
7. Tumor associated strokes was considered among patients with proven primary lesions with a histological diagnosis following either the therapeutic hematoma evacuation or minimally invasive biopsies.

**Figure 1.**
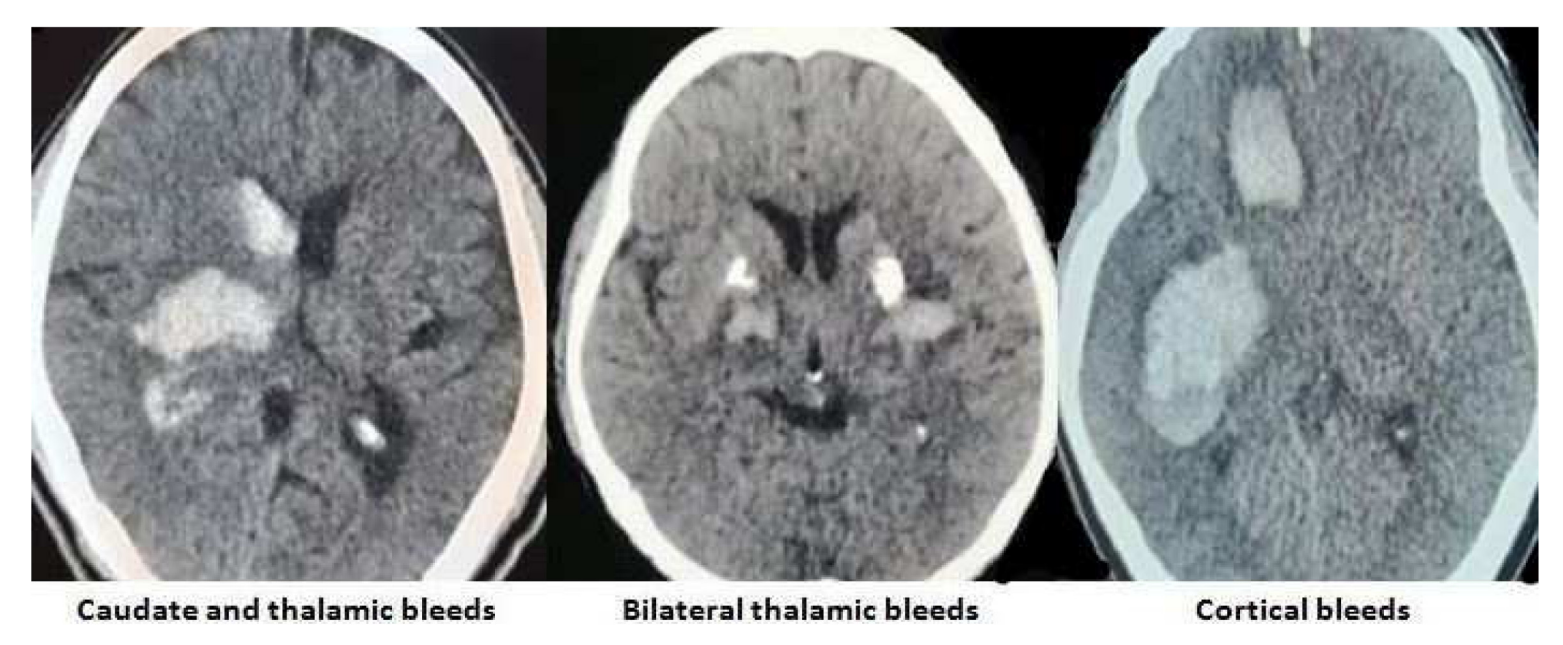
Hypertension and amyloid angiopathy as the cause of simultaneous multiple intracerebral hemorrhages. All images are original, deidentified, and of our patients themselves.

The images of the varied etiological causes of SMICH are demonstrated in the Figure 1– Figure 3.

**Figure 2.**
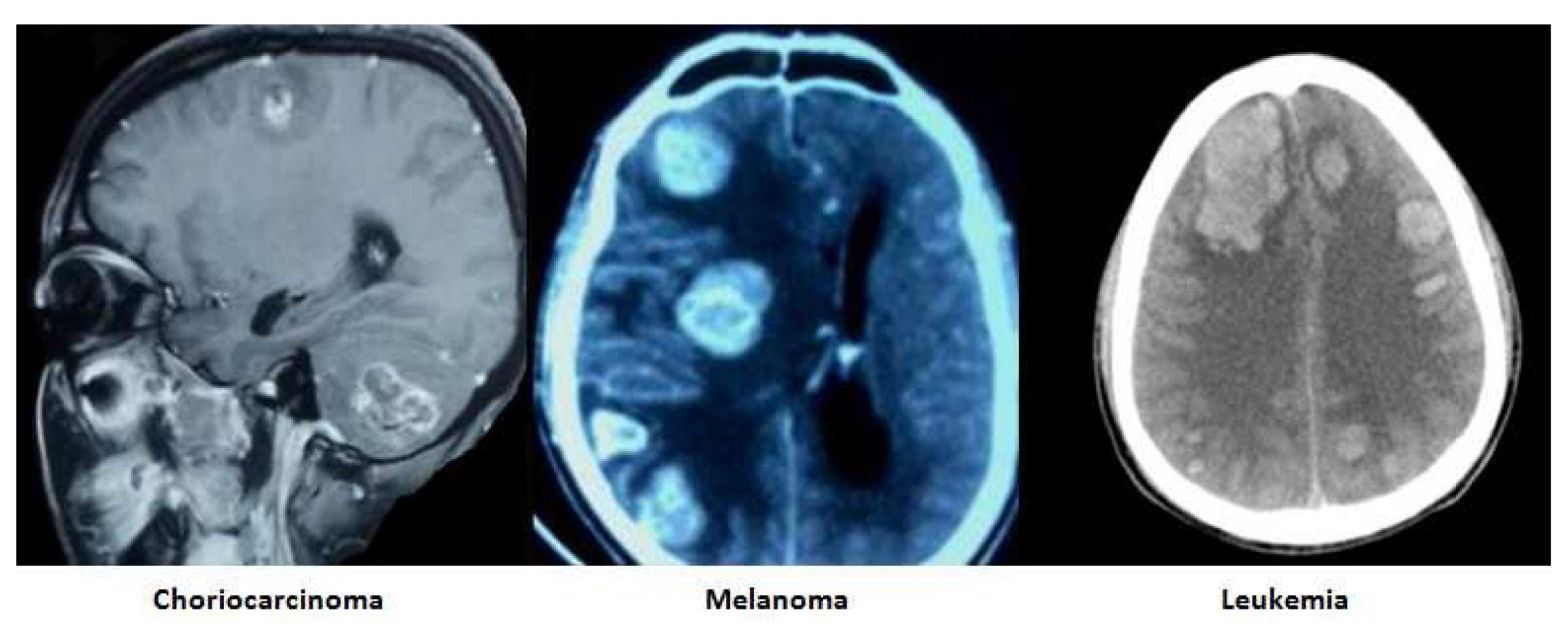
Tumor associated simultaneous multiple intracerebral hemorrhages. All images are original, deidentified, and of our patients themselves.

**Figure 3.**
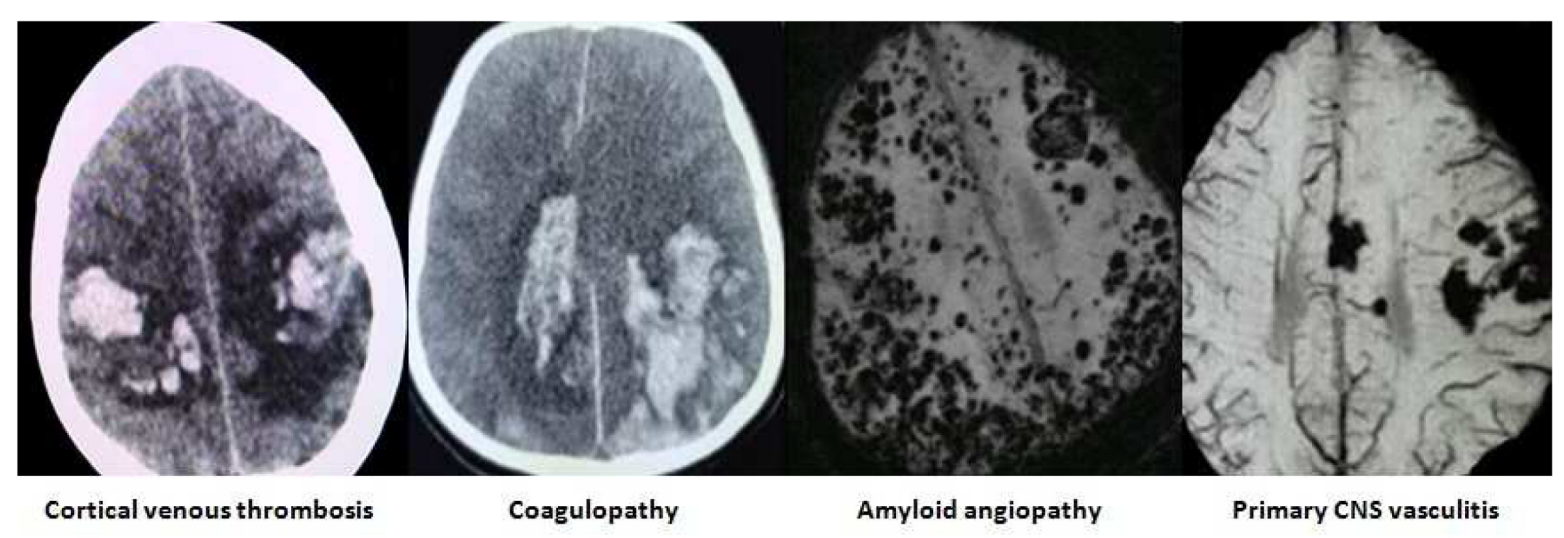
Miscellaneous etiological basis of simultaneous multiple intracerebral hemorrhages. All images are original, deidentified, and of our patients themselves.

### Regression analysis and hypothesis testing

The regression through analysis of variance (ANOVA) testing was adopted for examining the hypotheses formed as part of this research. Three hypotheses were tested and are analyzed in the following sections. The pertinent study variables of all 40 patients were retrieved and there were no missing data.

## Results

The patients’ demographics and clinico-radiological based outcomes are provided in Table 1 ( Munakomi, 2022).

**Table 1.** Analysis of our cohort presenting with simultaneous multiple intracerebral hemorrhages.

Study variables	Frequency (percentage)
**Etiological classification** • **Hypertension** • **Tumor** • **Amyloid angiopathy** • **Cortical venous thrombosis** • **Cavernoma** • **Anticoagulant** • **Vasculitis** • **Arteriovenous malformation**	• 9(22.5%) • 9(22.5%) • 8(20%) • 5(12.5%) • 3(7.5%) • 3(7.5%) • 2(5%) • 1(2.5%)
**Anatomical distribution** • **Cortical and cortical** • **Basal ganglion and thalamus** • **Brainstem and cerebellar** • **Cortical and cerebellar** • **Cortical and Basal ganglion** • **Basal ganglion and Basal ganglion** • **Brainstem and Brainstem** • **Cortical and Brainstem**	• 24(60%) • 5(12.5%) • 3(7.5%) • 2(5%) • 2(5%) • 2(5%) • 1(2.5%) • 1(2.5%)
**Outcome analysis** • **Conservative** • **Operative** • **Referral** • **Left against medical advice** • **Mortality**	• 15(37.5%) • 5(12%) • 6(15%) • 14(35%) • 5(12%)

### Demographic analysis

Among the cohort of 40 patients, the most common age group with 13(32.5%) patients was the 36–45 age group followed by 12(30%) in the 56–65 age group. The male to female gender ratio was 25:15 (62.5% vs. 37.5%).

### Risk, territorial and outcome analysis

The majority (75%) of the cohort had hypertension as the risk factor. Merely 10% of the respondents were affected by diabetes. The vast majority (97.5%) of patients lacked adherence to drug compliance. Cortical and cortical territory (60%) was the major anatomical patterns of involvement. Mortality was observed in 5 (12.5%) patients, and 14 (35%) patients left against medical advice. 

***Age and SMICH.*** Age related to SMICH was verified against the SMICH patients in the first hypothesis through the regression technique. From Table 2b, the Regression analysis (R (0.116), R2 (0.013) and Adjusted R2 (-0.012) were found; Table 2b represents that the ANOVA outcome of the age analysis is found as ‘insignificant’ (p=0.476).

The regression equation used in analyzing the association between SMICH and age in patients was calculated through:

SMICH = 3.692 + (-0.846 * AGE)

This equation is made from the beta values obtained from the above Table (2a)– Table (2c) after doing regression analysis.

**Table 2a.** Model Summary.

Model	Regression analysis (R)	Regression analysis (R ^2^)	Adjusted Regression analysis (R ^2^)	Standard Error of the Estimate	Change Statistics				
					Regression analysis (R ^2^) Change	Fischer (F) Change	Difference in fraction (f1)	Difference in fraction (f2)	Significant Fischer Change
1	0.116 ^a^	0.013	-0.012	1.15937	0.013	0.519	1	38	0.476

Predictors: (Constant), simultaneous multiple intracerebral hemorrhages Dependent Variable: Age

**Table 2b.** ANOVA.

Model		Sum of Squares	Difference in fraction (df)	Mean Square	Fischer (F)	Significance
1	Regression	0.698	1	0.698	0.519	0.476
	Residual	51.077	38	1.344		
	Total	51.775	39			

Predictors: (Constant), simultaneous multiple intracerebral hemorrhages Dependent Variable: Age

**Table 2c.** Coefficients.

Model		Un-standardized Coefficients		Standardized Coefficients	Significance
		Beta	Standard Error	Beta	
1	(Constant)	3.692	1.217		0.004
	SMICH	-0.846	1.174	-0.116	0.476

The outcome is negative denoting that the hypothesis is invalid where age doesn’t impact the likelihood of SMICH.

***Gender and SMICH.*** Gender was verified against the SMICH patients in the second hypothesis through the regression technique. From Table 3a, the R (0.207), R2 (0.043) and adjusted R2 (0.018) are shown; Table 3b suggests that the ANOVA outcome of the gender analysis is ‘insignificant’ (p=0.201).

**Table 3a.** Model Summary.

**Mode**	**Regression analysis (R)**	**Regression analysis (R ^2^)**	**Adjusted Regression analysis (R ^2^)**	**Standard Error of the Estimate**	**Change Statistics**				
					**Regression analysis (R ^2^) Change**	**Fischer Change**	**Difference in fraction (f1)**	**Difference in fraction (f2)**	**Significant Fischer Change**
1	0.207	0.043	0.018	0.48597	0.043	1.696	1	38	0.201

Predictors: (Constant), simultaneous multiple intracerebral hemorrhages Dependent variable: Gender

**Table 3b.** Analysis of variance.

Model		Sum of Squares	Difference in fraction (df)	Mean Square	Fischer test	Significance
1	Regression	0.401	1	0.401	1.696	0.201
	Residual	8.974	38	0.236		
	Total	9.375	39			

Dependent Variable: Gender Predictors: (Constant), simultaneous multiple intracerebral hemorrhages

Table 3c represents the association between SMICH patients and the gender factor. Regression equation in analyzing the SMICH patients and gender association was calculated through:

**Table 3c.** Coefficients.

Model		Un-standardized Coefficients		Standardized Coefficients	Significance
		Beta	Standard Error	Beta	
1	(Constant)	0.718	0.510		0.168
	SMICH	0.641	0.492	0.207	0.201

SMICH = 0.718 + (0.641 * GENDER)

This equation is made from the beta values obtained from the above Table (3a)– Table (3c) after doing regression analysis.

The outcome denotes that the hypothesis is invalid wherein gender doesn’t impact SMICH patients.

***Hypertension and SMICH.*** Hypertension as a factor affecting SMICH was verified, against the SMICH patients in the first hypothesis through the regression technique. From Table 4a, the R (0.000), R2 (0.000) and Adjusted R2 (0.000) were found; Table 4b represents that the ANOVA outcome of the hypertension analysis is found as ‘significant’ (p=0.000).

**Table 4a.** Model Summary.

Model	Regression analysis (R)	Regression analysis (R ^2^)	Adjusted Regression analysis (R ^2^)	Standard Error of the Estimate	Change Statistics				
					Regression analysis (R ^2^) Change	Fischer Change	Difference in fraction (df1)	Difference in fraction (df2)	Significant Fischer Change
1	0.160	0.026	0.000	0.50637	0.026	1.000	1	38	0.000

Predictors: (Constant), simultaneous multiple intracerebral hemorrhages Dependent variable: Hypertension

**Table 4b.** Analysis of variance.

Model		Sum of Squares	Difference in fraction (df)	Mean Square	Fischer test	Significance
1	Regression	0.256	1	0.256	1.000	0.000
	Residual	9.744	38	0.256		
	Total	10.000	39			

Dependent Variable: Hypertension Predictors: (Constant), simultaneous multiple intracerebral hemorrhages

Table 4c represents the association between SMICH patients and the hypertension. Regression equation in analyzing the SMICH patients and hypertension association was calculated through:

**Table 4c.** Coefficients.

Model		Un-standardized Coefficients		Standardized Coefficients	Gosset’s Student distribution (T)	Significance
		Beta	Standard Error	Beta		
1	(Constant)	2.026	0.532		3.810	0.000
	SMICH	-0.513	0.513	-0.160	-1.000	0.000

Dependent Variable: Hypertension

SMICH = .718 + (.641 * Hypertension)

This equation is made from the beta values obtained from the above Table (4a)– Table (4c) after doing regression analysis.

The outcome denotes that the hypothesis is valid where hypertension impacts SMICH patients.

## Discussion

In a study by Yen *et al.* (2005), the mean age of the patients presenting with SMICH was 60.6 ±7.9 years with a male:female ratio of 1.5:1.The mean age of similar cohorts in another study was 68.5 ± 12.8 years ( Yamaguchi *et al.*, 2017). Comparatively, our study saw much younger patients with 32.5% of patients in the age groups of 36–45 years. The male to female ratio in our study was 1.66:1.

Contrary to single ICH, SMICH significantly showed lobar territorial preponderance ( Wu *et al.*, 2017) ( Renard *et al.*, 2020). Our study had cortical-cortical patterns of involvement in 60% of the patients and basal ganglion and thalamic involvement in 12.5% of cases. Putamen and thalamus patterns of involvement were the most common anatomical patterns of involvement in a study by Yamaguchi *et al.* (2017) whereas bilateral thalamic hemorrhages variants were the most common in a study by Yen *et al.* (2005).

The indication of surgery among patients with SMICH depends upon variables such as presenting GCS, location and size of the hematomas ( Yi *et al.*, 2013). Surgery has been advocated among patients with lobar or cerebellar hematomas ( Yen *et al.*, 2005). In a study by Yamaguchi *et al.* (2017), 35.3% of patients underwent craniotomy. Surgery was performed in only 12% of our cohort.

There are variables connoting clinical outcome among patients with SMICH. One study found low Glasgow Coma Scale (GCS) results, large hematoma and concurrent ventricular extension to be prognostic markers determining 90-day mortality ( Wu *et al.*, 2017). Unilateral supratentorial hematomas showed the most favorable outcomes ( Zaorsky *et al.*, 2019), while deep seated SMICH had the highest mortality ( Renard *et al.*, 2020). The SMICH mortality described in the literature is high, ranging from 37% to 50% ( Wu *et al.*, 2017) ( Yen *et al.*, 2005). The mortality in our study was comparatively low at 12%. 15% of the patients in our study were referred to appropriate oncology center while 35% of them left despite medical advice.

Hypertensive angiopathy and cerebral amyloid angiopathy accounted for more than 50% of cases in SMICH (Wu TY *et al.*) ( Renard *et al.*, 2020) ( Stemer *et al.*, 2010). They accounted for only 42.5% of the SMICH in our study. In total, 75% of our patients presenting with SMICH had a history of hypertension and were hypertensive on presentation. Paradoxically, 97.5% of diagnosed cases of hypertension lacked compliance to their medications.

The pathophysiology of SMICH was proposed to be due to degenerative alterations in the intraparenchymal arterioles caused by long-term, uncontrolled hypertension and the simultaneous rupture of bilateral charcot-bouchard microaneurysms ( Yen *et al.*, 2005). As per the “biphasic hypothetical mechanism," an initial ictal hematoma causes an adrenaline spike that disrupts the cerebral autoregulation thereby harbingering the rupture of already weakened arterioles ( Yen *et al.*, 2005). Primary SMICH characteristically showed high microhemorrhages burden of varying age in MRI studies. This also highlights the probable role of auto-regulatory dysfunction following the ictal bleed as the prime pathological genesis behind the SMICH ( Stemer *et al.*, 2010).

This real-world incidence, patterns and outcome of SMICH can only be validated following a nation-wide multicenter database study. Due to the location and specificity of our results they may not be generalizable to patients from other ethnic and geographic backgrounds. The recall bias (about the risk factors and the medical compliance by the patients when the information was provided by their kin) can be another limiting issue in this observational study.

## Conclusions

This is one of the first observational studies pertaining to SMICH to be carried out within our subcontinent. This study proves the need fora national stroke data bank pertaining to SMICH. This will help foster effective patient education during preoperative counseling; as well as formatting a management algorithm combating them.

## Data availability

### Underlying data

Figshare: Simultaneous multiple intracerebral hemorrhages. https://doi.org/10.6084/m9.figshare.19063982.v2 ( Munakomi, 2022).

This project contains the following underlying data:

- SIMS deep.xlsx (de-identified raw medical data)

Data are available under the terms of the Creative Commons Attribution 4.0 International license (CC-BY 4.0).
