# Demographical domains and clinico-radiological characteristics of study cohorts with spontaneous simultaneous multiple intracerebral hemorrhages in a tertiary neurosurgical center in Nepal: a cross-sectional study

**DOI:** 10.12688/f1000research.108816.2

**Published:** 2022-11-28

**Authors:** Sunil Munakomi, Dipak Chaulagain

**Affiliations:** 1 Neurosurgery, College of Medical Sciences, Chitwan, Nepal; 2 Neurosurgery, Uzhhorod National University, Uzhhorod, Ukraine

**Keywords:** multiple strokes, simultaneous strokes, cause, patterns, pathogenesis, outcome

## Abstract

**Background:** Spontaneous simultaneous multiple intra-cerebral hemorrhages (SMICHs) and its occurrences in different territories of arterial disposition has been viewed as uncommon clinical occurrences, since the pathophysiological and predisposing factors as mechanisms aren’t vividly defined.

This research primarily aims for demographic stratification and dichotomization pertaining to risk factors, etiological classifications, anatomical distributions and outcome analysis by focusing on management strategies and pertinent stroke care.

**Methods: **40 patients presenting to the College of Medical Sciences, Chitwan, Nepal in the last two years were included in the study. The patients with two or more spontaneous SMICHs with affected arterial territories with similar tomographic density based profiling were chosen as samples. Regression analysis was chosen to test three hypotheses.

**Results:** Among our study cohorts, cortical and cortical territory (60%) was the major anatomical patterns of involvement. A conservative approach was undertaken in nine patients (22.5%), whereas surgical intervention was needed in five others (12.5%). A total of 14(35%) patients leaving against medical advice and a further seven (17.5%) patients were referred for adjuvant oncologic care. Mortality was observed among five (12.5%) patients. Hypertension was seen as a significant variable in its pathogenesis. Male patients were more affected. Age groups comprising 36-45years and 56-65 years were involved in 32.5% and 30% of cases respectively.

**Conclusion:** This study proves the need for a national stroke data bank pertaining to spontaneous SMICHs. This will help foster effective patient education during preoperative counseling; as well as formatting a management algorithm combating them.

## Introduction

Spontaneous simultaneous multiple intra-cerebral hemorrhages (SMICHs) are characterized by intracerebral hemorrhages within the two-or-more distinct and non-contiguous intracranial vascular territories visualized in the initial radiological imaging ( Wu *et al.*, 2017). Generally intracerebral hemorrhage (ICH) connotes high odds of continuum morbidity and mortality ( Laiwattana *et al.*, 2014). Among ICH patients, approximately 5% (1:20) have been identified as SMICHs ( Renard *et al.*, 2020).

Stroke ripples affect patients with continuum multi-spectral consequences ( Gokce *et al.*, 2016). Literature scarcity pertaining to spontaneous SMICHs paves the way for further research on the entity. Better knowledge and in-depth information pertaining to the incidence and patterns of spontaneous SMICHs can help formulate treatment algorithms as well as provide newer reforms in patient management system.

### Hypotheses

The research focused on pattern analysis on how the patients with spontaneous SMICHs are affected by applying age and gender as demographic variable and “hypertension” as the major risk variable.

Thus, three hypotheses were formulated:

– ***Hypothesis 1:*** There exists an imperative association between age and spontaneous SMICHs;
– ***Hypothesis 2:*** There exists an imperative association between gender and spontaneous SMICHs;
– ***Hypothesis 3:*** There exists an imperative association between the hypertension and spontaneous SMICHs.

### Rationale of study

The primary aim was to examine risk factors in spontaneous SMICHs patients:

– To analyze the demographic profile (age and gender) of spontaneous SMICHs patients;
– To analyze pivotal factors that impact spontaneous SMICHs patients significantly, namely: hypertension, diabetes, etiological classifications, anatomical distributions and outcome analysis.

## Methods

The methodology for the research was focused to spontaneous SMICHs patients from the hospital medical record section of the College of Medical Sciences, in Bharatpur, Chitwan, Nepal, presenting between January 2019 to January 2021 (time we started recording and compiling data of patients with spontaneous SMICHs). The outcomes were compiled to study the patterns through frequencies measures and analysis. The approval for the research conduction was obtained by the Institutional Review Committee (COMSTH-IRC/2021–56). Written informed consent was obtained from all the participants or their next of kin (in case their poor Glasgow coma scale did not allow taking consent from the patients themselves) at the time of their admissions.

### Theoretical framework

The research mainly focused upon the spontaneous SMICHs patients as the independent variable and how it impacts the respondents under varied dependent variables like: age, gender and hypertension.

### Inclusion and exclusion criteria

40 patients from the College of Medical Sciences, Chitwan, Nepal with spontaneous SMICHs were included in this study. Data was retrieved from the medical record section of the hospital pertaining to patients presenting with spontaneous SMICHs. The study size was arrived through convenience sampling.

Inclusion criteria

- All patients presenting with spontaneous SMICHs.

Exclusion criteria include:

- History of previous strokes;
- Patients with recurrent strokes;
- Failure to obtain consent for participation in the study.

### Target and sampling

The minimal necessary sampling for research has been estimated by Fischer’s formula ( Charan & Biwas, 2013) as

N=z ^2^ x p x q/d ^2^

Where:

***z*** represents a value of *1.96* at the confidence-interval of 95%,

***p*** represents SSMICH prevalence as 6%,

***q*** as 1-p and

***d*** represents the margin-of-error as 10%.

The final result through sampling estimation was calculated as 21.66 and the sample-size of 40 patients was finalized for the research purpose.

### Variables under focus

Spontaneous SMICHs is the presence of two-or-more ICHs that affects different vascular territories with similar tomographic density-profiles. All findings were validated with radiologists.

### Data collection

All data was taken from patient medical records.

Etiologic classification for the cause of ICH was stratified as per the SMASH-U classification into hypertensive angiopathy, cerebral amyloid angiopathy, structural vascular abnormalities, medication related, other systemic causes, and undefined causes respectively ( Mosconi *et al.*, 2021). The risk factors were defined as:

1. Arterial hypertension if blood pressure was above 160/90 mm Hg for more than two readings at the time of admission, on antihypertensive drugs or previous medical history of hypertension.
2. Diabetes mellitus if fasting glucose level was >120 mg/dL at the time of admission; hypercholesterolemia, and fasting cholesterol level >220 mg/dL.
3. Vascular lesion with pertinent radiological imaging at the site of the ICH was classified as the primary cause of the hematoma.
4. Cerebral venous sinus thrombosis was diagnosed on a cerebral venogram.
5. Drug associated ICH was considered in a patient on warfarin with international normalized ratio ≥2.0, novel oral anticoagulants within three days, full-dose heparin, or on systemic thrombolysis.
6. Cerebral amyloid angiopathy was classified in patients’ ≥55 years of age with predominant lobar bleeds with microbleeds on magnetic resonance imaging (MRI) sequences sparing the basal ganglion and the thalamus.
7. Tumor associated strokes was considered among patients with proven primary lesions with a histological diagnosis following either the therapeutic hematoma evacuation or minimally invasive biopsies.

All patients were thoroughly evaluated for all possible etiological factors including cerebral angiography (including the venogram) and MRI stroke protocol (T1/T2/FLAIR/DWI/SWI/MR angiography) whenever feasible. The biopsy of the hematoma was sent for every operative case so as to aid in the definitive cause of the SMICHs. 

**Figure 1.**
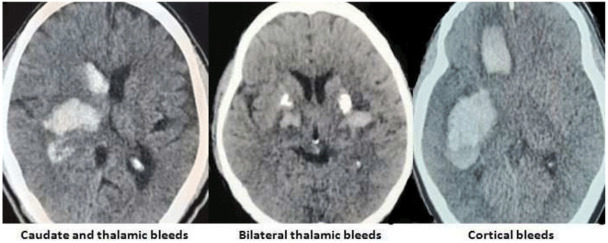
Hypertension and amyloid angiopathy as the cause of spontaneous simultaneous multiple intracerebral hemorrhages. All images are original, deidentified, and of our patients themselves.

The images of the varied etiological causes of SMICH are demonstrated in the Figure 1– Figure 3.

**Figure 2.**
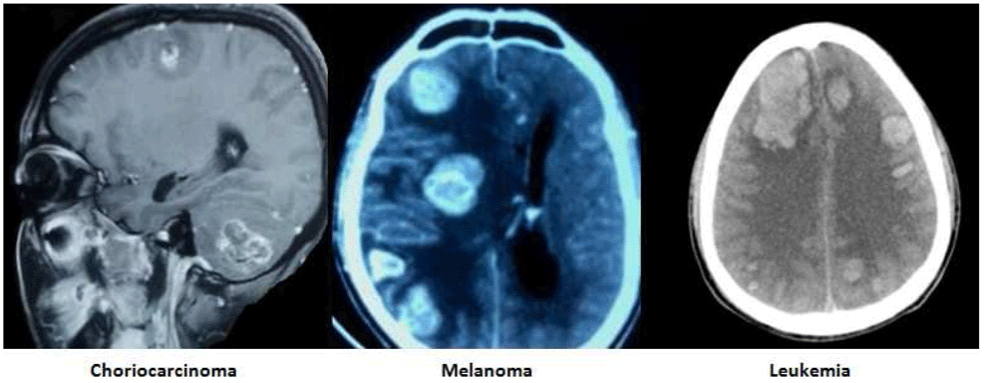
Tumor associated spontaneous simultaneous multiple intracerebral hemorrhages. All images are original, deidentified, and of our patients themselves.

**Figure 3.**
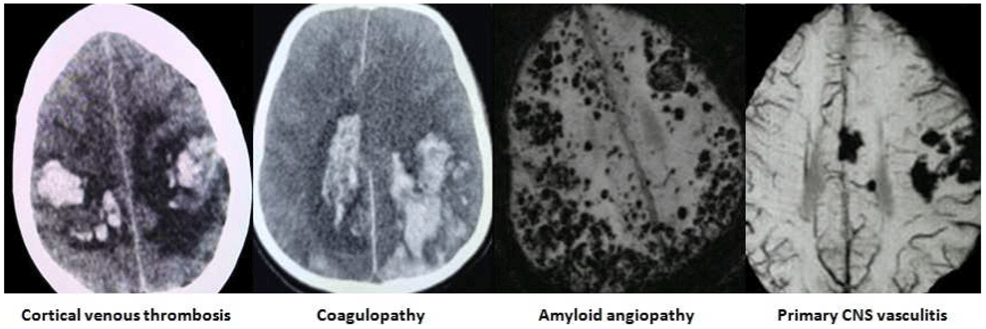
Miscellaneous etiological basis of spontaneous simultaneous multiple intracerebral hemorrhages. All images are original, deidentified, and of our patients themselves.

### Regression analysis and hypothesis testing

The regression through analysis of variance (ANOVA) testing was adopted for examining the hypotheses formed as part of this research. Three hypotheses were tested and are analyzed in the following sections. The pertinent study variables of all 40 patients were retrieved and there were no missing data.

## Results

The patients’ demographics and clinico-radiological based outcomes are provided in Table 1.

**Table 1.** Analysis of our cohort presenting with spontaneous simultaneous multiple intracerebral hemorrhages.

Study variables	Frequency (percentage)
**Etiological classification** • **Hypertension** • **Tumor** • **Amyloid angiopathy** • **Cortical venous thrombosis** • **Cavernoma** • **Anticoagulant** • **Vasculitis** • **Arteriovenous malformation**	• 9(22.5%) • 9(22.5%) • 8(20%) • 5(12.5%) • 3(7.5%) • 3(7.5%) • 2(5%) • 1(2.5%)
**Anatomical distribution** • **Cortical and cortical** • **Basal ganglion and thalamus** • **Brainstem and cerebellar** • **Cortical and cerebellar** • **Cortical and Basal ganglion** • **Basal ganglion and Basal ganglion** • **Brainstem and Brainstem** • **Cortical and Brainstem**	• 24(60%) • 5(12.5%) • 3(7.5%) • 2(5%) • 2(5%) • 2(5%) • 1(2.5%) • 1(2.5%)
**Outcome analysis** • **Conservative** • **Operative** • **Referral** • **Left against medical advice** • **Mortality**	• 15(37.5%) • 5(12%) • 6(15%) • 14(35%) • 5(12%)

### Demographic analysis

Among the cohort of 40 patients, the most common age group with 13(32.5%) patients was the 36–45 age group followed by 12(30%) in the 56–65 age group. The male to female gender ratio was 25:15 (62.5% vs. 37.5%).

### Risk, territorial and outcome analysis

The incidence of spontaneous SMICHs from our study (40 cases) within the specified duration of two years (total 250 cases) was 16%. The majority (75%) of the cohort had hypertension as the risk factor. Merely 10% of the respondents were affected by diabetes. The vast majority (97.5%) of patients lacked adherence to drug compliance. Cortical and cortical territory (60%) was the major anatomical patterns of involvement. Mortality was observed in 5 (12.5%) patients, and 14 (35%) patients left against medical advice. Surgery was required in 12% of the cohorts who presented with cortical, subcortical and cerebellar bleeds and had clinical features of cerebral herniation. Surgery was aimed to remove the clinically significant hematomas only. One mortality (20%) was observed among the individuals who underwent operative intervention. Ther overall mortality in the study was 12%.

***Age and spontaneous SMICHs.*** Age related to spontaneous SMICHs was verified against the spontaneous SMICHs patients in the first hypothesis through the regression technique. From Table 2b, the Regression analysis (R (0.116), R2 (0.013) and Adjusted R2 (-0.012) were found; Table 2b represents that the ANOVA outcome of the age analysis is found as ‘insignificant’ (p=0.476).

The regression equation used in analyzing the association between spontaneous SMICHs and age in patients was calculated through:

Spontaneous SMICHs = 3.692 + (-0.846 * AGE)

This equation is made from the beta values obtained from the above Table (2a)– Table (2c) after doing regression analysis.

**Table 2a.** Model Summary.

Model	Regression analysis (R)	Regression analysis (R ^2^)	Adjusted Regression analysis (R ^2^)	Standard Error of the Estimate	Change Statistics				
					Regression analysis (R ^2^) Change	Fischer (F) Change	Difference in fraction (f1)	Difference in fraction (f2)	Significant Fischer Change
1	0.116 ^a^	0.013	-0.012	1.15937	0.013	0.519	1	38	0.476

Predictors: (Constant), spontaneous simultaneous multiple intracerebral hemorrhages Dependent Variable: Age

**Table 2b.** ANOVA.

Model		Sum of Squares	Difference in fraction (df)	Mean Square	Fischer (F)	Significance
1	Regression	0.698	1	0.698	0.519	0.476
	Residual	51.077	38	1.344		
	Total	51.775	39			

Predictors: (Constant), spontaneous simultaneous multiple intracerebral hemorrhages Dependent Variable: Age

**Table 2c.** Coefficients.

Model		Un-standardized Coefficients		Standardized Coefficients	Significance
		Beta	Standard Error	Beta	
1	(Constant)	3.692	1.217		0.004
	SSMICH	-0.846	1.174	-0.116	0.476

The outcome is negative denoting that the hypothesis is invalid where age doesn’t impact the likelihood of spontaneous SMICHs.

***Gender and spontaneous SMICHs.*** Gender was verified against the spontaneous SMICHs patients in the second hypothesis through the regression technique. From Table 3a, the R (0.207), R2 (0.043) and adjusted R2 (0.018) are shown; Table 3b suggests that the ANOVA outcome of the gender analysis is ‘insignificant’ (p=0.201).

**Table 3a.** Model Summary.

**Mode**	**Regression analysis (R)**	**Regression analysis (R ^2^)**	**Adjusted Regression analysis (R ^2^)**	**Standard Error of the Estimate**	**Change Statistics**				
					**Regression analysis (R ^2^) Change**	**Fischer Change**	**Difference in fraction (f1)**	**Difference in fraction (f2)**	**Significant Fischer Change**
1	0.207	0.043	0.018	0.48597	0.043	1.696	1	38	0.201

Predictors: (Constant), spontaneous simultaneous multiple intracerebral hemorrhages Dependent variable: Gender

**Table 3b.** Analysis of variance.

Model		Sum of Squares	Difference in fraction (df)	Mean Square	Fischer test	Significance
1	Regression	0.401	1	0.401	1.696	0.201
	Residual	8.974	38	0.236		
	Total	9.375	39			

Dependent Variable: Gender Predictors: (Constant), spontaneous simultaneous multiple intracerebral hemorrhages

Table 3c represents the association between spontaneous SMICHs patients and the gender factor. Regression equation in analyzing the spontaneous SMICHs patients and gender association was calculated through:

**Table 3c.** Coefficients.

Model		Un-standardized Coefficients		Standardized Coefficients	Significance
		Beta	Standard Error	Beta	
1	(Constant)	0.718	0.510		0.168
	Spontaneous SMICHs	0.641	0.492	0.207	0.201

Spontaneous SMICHs = 0.718 + (0.641 * GENDER)

This equation is made from the beta values obtained from the above Table (3a)– Table (3c) after doing regression analysis.

The outcome denotes that the hypothesis is invalid wherein gender doesn’t impact spontaneous SMICHs patients.

***Hypertension and spontaneous SMICHs.*** Hypertension as a factor affecting spontaneous SMICHs was verified, against the spontaneous SMICHs patients in the first hypothesis through the regression technique. From Table 4a, the R (0.000), R2 (0.000) and Adjusted R2 (0.000) were found; Table 4b represents that the ANOVA outcome of the hypertension analysis is found as ‘significant’ (p=0.000).

**Table 4a.** Model Summary.

Model	Regression analysis (R)	Regression analysis (R ^2^)	Adjusted Regression analysis (R ^2^)	Standard Error of the Estimate	Change Statistics				
					Regression analysis (R ^2^) Change	Fischer Change	Difference in fraction (df1)	Difference in fraction (df2)	Significant Fischer Change
1	0.160	0.026	0.000	0.50637	0.026	1.000	1	38	0.000

Predictors: (Constant), spontaneous simultaneous multiple intracerebral hemorrhages Dependent variable: Hypertension

**Table 4b.** Analysis of variance.

Model		Sum of Squares	Difference in fraction (df)	Mean Square	Fischer test	Significance
1	Regression	0.256	1	0.256	1.000	0.000
	Residual	9.744	38	0.256		
	Total	10.000	39			

Dependent Variable: Hypertension Predictors: (Constant), spontaneous simultaneous multiple intracerebral hemorrhages

Table 4c represents the association between spontaneous SMICHs patients and the hypertension. Regression equation in analyzing the spontaneous SMICHs patients and hypertension association was calculated through:

**Table 4c.** Coefficients.

Model		Un-standardized Coefficients		Standardized Coefficients	Gosset’s Student distribution (T)	Significance
		Beta	Standard Error	Beta		
1	(Constant)	2.026	0.532		3.810	0.000
	Spontaneous SMICHs	-0.513	0.513	-0.160	-1.000	0.000

Dependent Variable: Hypertension

Spontaneous SMICHs = .718 + (.641 * Hypertension)

This equation is made from the beta values obtained from the above Table (4a)– Table (4c) after doing regression analysis.

The outcome denotes that the hypothesis is valid where hypertension impacts spontaneous SMICHs patients.

While comparing to the cohorts of 85 patients with single spontaneous intra-cerebral hemorrhage (ICH) that underwent operative intervention in our institution during the same interval, the male: female ratio was 1.66:1 compared to 4.3:1 for spontaneous SMICHs ( Bhattarai *et al.*, 2021). The history of hypertension and compliance to medication was observed in 83.5% and 35.5% respectively compared to 75% and 2.5% among patients with spontaneous SMICHs. The single spontaneous ICH had the predominant involvement of the basal ganglionic compared to the cortical territory at the ratio of 3:1. The cortical and cortical patterns of involvement were observed in 60% of cohorts with spontaneous SMICHs. The mortality was 3.8% in operative patients for single ICH compared to 20% among cohorts who underwent operative intervention for spontaneous SMICHs.

## Discussion

The incidence of spontaneous SMICHs from our study (40 cases) within the specified duration of two years (total 250 cases) in our institution was 16%. In a study by Yen *et al.* (2005), the mean age of the patients presenting with SSMICH was 60.6 ±7.9 years with a male:female ratio of 1.5:1. The mean age of similar cohorts in another study was 68.5 ± 12.8 years ( Yamaguchi *et al.*, 2017). Comparatively, our study saw much younger patients with 32.5% of patients in the age groups of 36–45 years. The male to female ratio in our study was 1.66:1.

Contrary to single ICH, spontaneous SMICHs significantly showed lobar territorial preponderance ( Wu *et al.*, 2017) ( Renard *et al.*, 2020). Our study had cortical-cortical patterns of involvement in 60% of the patients and basal ganglion and thalamic involvement in 12.5% of cases. Putamen and thalamus patterns of involvement were the most common anatomical patterns of involvement in a study by Yamaguchi *et al.* (2017) whereas bilateral thalamic hemorrhages variants were the most common in a study by Yen *et al.* (2005).

The indication of surgery among patients with spontaneous SMICHs depends upon variables such as presenting GCS, location and size of the hematomas ( Yi *et al.*, 2013). Surgery has been advocated among patients with lobar or cerebellar hematomas ( Yen *et al.*, 2005). In a study by Yamaguchi *et al.* (2017), 35.3% of patients underwent craniotomy. Surgery was performed in only 12% of our cohort.

There are variables connoting clinical outcome among patients with spontaneous SMICHs. One study found low Glasgow Coma Scale (GCS) results, large hematoma and concurrent ventricular extension to be prognostic markers determining 90-day mortality ( Wu *et al.*, 2017). Unilateral supratentorial hematomas showed the most favorable outcomes ( Zaorsky *et al.*, 2019), while deep seated spontaneous SMICHs had the highest mortality ( Renard *et al.*, 2020). The spontaneous SMICHs mortality described in the literature is high, ranging from 37% to 50% ( Wu *et al.*, 2017) ( Yen *et al.*, 2005). The mortality in our study was comparatively low at 12%. 15% of the patients in our study were referred to appropriate oncology center while 35% of them left despite medical advice.

Hypertensive angiopathy and cerebral amyloid angiopathy accounted for more than 50% of cases in spontaneous SMICHs (Wu TY *et al.*) ( Renard *et al.*, 2020) ( Stemer *et al.*, 2010). They accounted for only 42.5% of the spontaneous SMICHs in our study. In total, 75% of our patients presenting with spontaneous SMICHs had a history of hypertension and were hypertensive on presentation. Paradoxically, 97.5% of diagnosed cases of hypertension lacked compliance to their medications.

The pathophysiology of spontaneous SMICHs was proposed to be due to degenerative alterations in the intraparenchymal arterioles caused by long-term, uncontrolled hypertension and the simultaneous rupture of bilateral charcot-bouchard microaneurysms ( Yen *et al.*, 2005). As per the “biphasic hypothetical mechanism," an initial ictal hematoma causes an adrenaline spike that disrupts the cerebral autoregulation thereby harbingering the rupture of already weakened arterioles ( Yen *et al.*, 2005). Primary spontaneous SMICHs characteristically showed high microhemorrhages burden of varying age in MRI studies. This also highlights the probable role of auto-regulatory dysfunction following the ictal bleed as the prime pathological genesis behind the spontaneous SMICHs ( Stemer *et al.*, 2010).

While reviewing the study conducted in our institution, cohorts with spontaneous SMICHs had higher male preponderance, poor compliance to antihypertensive therapy, predominant cortical involvement and increased operative mortality when compared to cohorts undergoing operative intervention for single spontaneous ICH ( Bhattarai *et al.*, 2021). 

This real-world incidence, patterns and outcome of spontaneous SMICHs can only be validated following a nation-wide multicenter database study. Due to the location and specificity of our results they may not be generalizable to patients from other ethnic and geographic backgrounds. The recall bias (about the risk factors and the medical compliance by the patients when the information was provided by their kin) can be another limiting issue in this observational study.

## Conclusions

This is one of the first observational studies pertaining to spontaneous SMICHs to be carried out within our subcontinent. This study proves the need for a national stroke data bank pertaining to spontaneous SMICHs. This will help foster effective patient education during preoperative counseling; as well as formatting a management algorithm combating them.

## Data Availability

Figshare: Simultaneous multiple intracerebral hemorrhages. https://doi.org/10.6084/m9.figshare.19063982.v2 ( Munakomi, 2022). This project contains the following underlying data: SIMS deep.xlsx (de-identified raw medical data) Data are available under the terms of the Creative Commons Attribution 4.0 International license (CC-BY 4.0).
