# Supplementary material for: Demographical domains and clinico-radiological characteristics of study cohorts with spontaneous simultaneous multiple intracerebral hemorrhages in a tertiary neurosurgical center in Nepal: a cross-sectional study
Source: F1000Res. 2022 Nov 28;11:154. Originally published 2022 Feb 7. [Version 2] doi: 10.12688/f1000research.108816.2 (PMC9716112; doi:10.12688/f1000research.108816.2)
Supplement: Peer review history

# Peer review: Reviewer response for version 2

**DOI:** 10.5256/f1000research.141319.r156807

**Published:** 2022-12-01

**Authors:** Amit Thapa

**Affiliations:** 1 Department of Neurological Surgery, Kathmandu Medical College And Teaching Hospital, Kathmandu, Nepal

The authors have revised the article suitably. The 16% incidence rate of SMICH is considerable. SMICH has been found increasingly in younger age males over cortical areas with non-compliance on anti hypertensive drugs and required more surgical intervention than isolated bleed. These facts are to be highlighted in conclusion.

Is the work clearly and accurately presented and does it cite the current literature?

Yes

If applicable, is the statistical analysis and its interpretation appropriate?

Yes

Are all the source data underlying the results available to ensure full reproducibility?

Yes

Is the study design appropriate and is the work technically sound?

Yes

Are the conclusions drawn adequately supported by the results?

Partly

Are sufficient details of methods and analysis provided to allow replication by others?

Yes

Reviewer Expertise:

Neurovascular

I confirm that I have read this submission and believe that I have an appropriate level of expertise to confirm that it is of an acceptable scientific standard.

# Peer review: Reviewer response for version 1

**DOI:** 10.5256/f1000research.120245.r153191

**Published:** 2022-11-14

**Authors:** Amit Thapa

**Affiliations:** 1 Department of Neurological Surgery, Kathmandu Medical College And Teaching Hospital, Kathmandu, Nepal

Authors have studied 40 patients over a period of two years with spontaneous simultaneous multiple intra-cerebral hemorrhages (SMICHs). Being not so common, this entity demands more elaborate study.

 The present study evaluates the association of age, gender and presence of hypertension with SMICHs. Authors by using SMASH-U classification have simplified as well as standardized the etiological classification of ICH in this study.

- It is suggested to add “spontaneous” to SMICHs to differentiate from traumatic conditions.
- The formula for calculating sample size needs to be formatted to convey the correct method of identifying minimal necessary sample size.
- It is pertinent to mention how the authors classified patients with mixed etiologies or when two or more factors exist without fulfilling the definition individually.
- To identify the etiological factors, what were all investigations carried on each patient?
- Table 1 when mentioned in the text, is presented with a reference to figshare dataset. These two are however are different. This confuses the reader. It would be good to mention the dataset separately as done in the "data availability" section.
- Minor corrections like adjusting font size and style are required to allow proper spacing between the words and letters.
- Besides the above, it would have been great if the authors compared SMICHs with single ICH for the variables they have studied, from the available dataset. The incidence of SMICH among all ICH could also have been calculated in Nepalese cohort.
- The fact that various researchers have found varied anatomical distribution of SMICHs demonstrate no territorial predilection for SMICHs.
- Results do not talk about who needed surgical treatment for SMICH and their outcome. However these datasets are discussed in Discussion.

Overall the authors need to be commended to have properly conducted and presented nicely the findings of this little researched condition.

Is the work clearly and accurately presented and does it cite the current literature?

Yes

If applicable, is the statistical analysis and its interpretation appropriate?

Yes

Are all the source data underlying the results available to ensure full reproducibility?

Yes

Is the study design appropriate and is the work technically sound?

Yes

Are the conclusions drawn adequately supported by the results?

Partly

Are sufficient details of methods and analysis provided to allow replication by others?

Yes

Reviewer Expertise:

Neurovascular

I confirm that I have read this submission and believe that I have an appropriate level of expertise to confirm that it is of an acceptable scientific standard, however I have significant reservations, as outlined above.

# Peer review: Reviewer response for version 1

**DOI:** 10.5256/f1000research.120245.r122721

**Published:** 2022-03-07

**Authors:** Guo-Yi Gao

**Affiliations:** 1 Department of Neurosurgery, Shanghai General Hospital, Shanghai Jiao Tong University School of Medicine, Shanghai, China

This study described the clinical features of simultaneous multiple intracerebral hemorrhages in a neurosurgical center in Nepal. The study was well designed and the data acquisition and analysis are both reasonable. The limitation, such as the sample size may influence the results, could be emphasized at the end of the manuscript thus to avoid an inadequate citing of the results in any other scenarios.

Is the work clearly and accurately presented and does it cite the current literature?

Yes

If applicable, is the statistical analysis and its interpretation appropriate?

Yes

Are all the source data underlying the results available to ensure full reproducibility?

Yes

Is the study design appropriate and is the work technically sound?

Yes

Are the conclusions drawn adequately supported by the results?

Yes

Are sufficient details of methods and analysis provided to allow replication by others?

Yes

Reviewer Expertise:

TBI

I confirm that I have read this submission and believe that I have an appropriate level of expertise to confirm that it is of an acceptable scientific standard.
